# Supplementary material for: IFN-Gamma and TNF-Alpha as a Priming Strategy to Enhance the Immunomodulatory Capacity of Secretomes from Menstrual Blood-Derived Stromal Cells
Source: Int J Mol Sci. 2021 Nov 10;22(22):12177. doi: 10.3390/ijms222212177 (PMC8618369; doi:10.3390/ijms222212177)
Supplement: Supplementary file 1 [file ijms-22-12177-s001.zip › ijms-1453057-supplementary.pdf]

Supplementary Materials

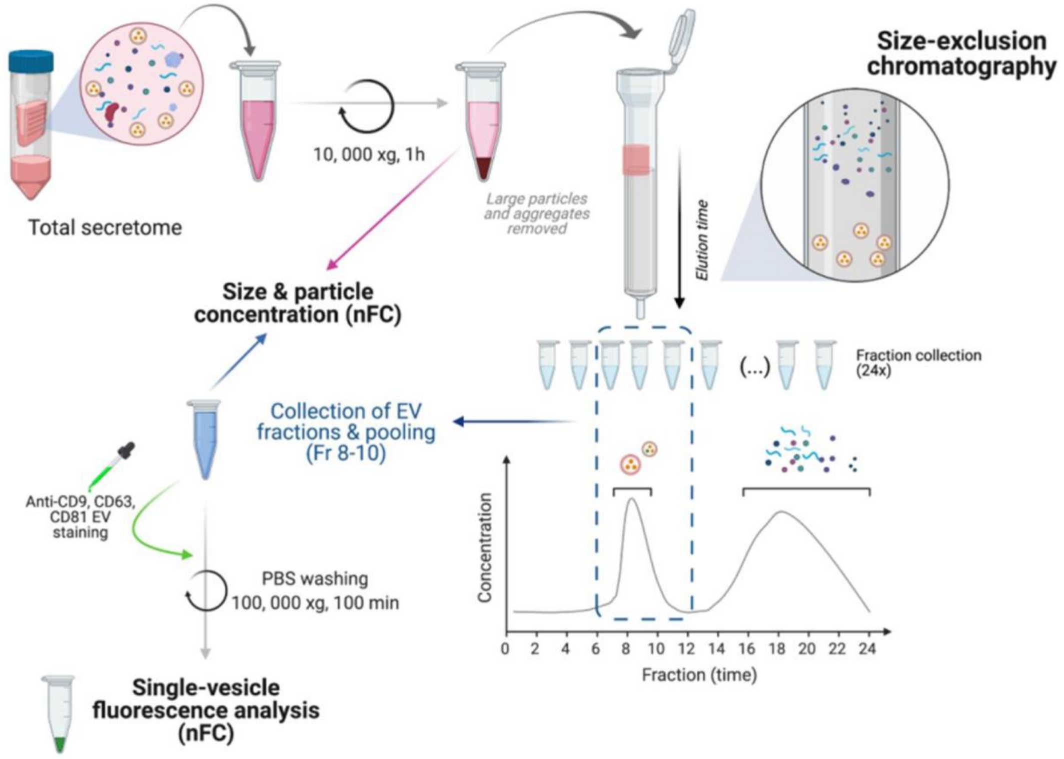

Figure S1. Methodological workflow for the analysis of the EVs compartment in MenSC secretomes. Total secretome samples were centrifuged at 10,000 × g for 1 h to remove large particles and aggregates. In the following, size and particle concentration were determined by nFC. To enrich the EVs and to separate them from the soluble protein fraction, size-exclusion chromatography (SEC) was performed. A total of 24 fractions were collected in which EVs were enriched in fractions 8–10. EV fractions were pooled and size and particle concentration were again measured by nFC. Finally, EV pools (similar number of particles between conditions) were stained with anti-CD9, CD63, and CD81 FITC-coupled antibodies. Before analyses, the non-labeled antibody was removed by PBS washing and centrifugation at 100,000 × g for 2 h, at 4 °C. Stained EV-pellets were suspended in PBS and analyzed by nFC according to the manufacturer´s instructions. This figure has been created with BioRender (https://app.biorender.com/).

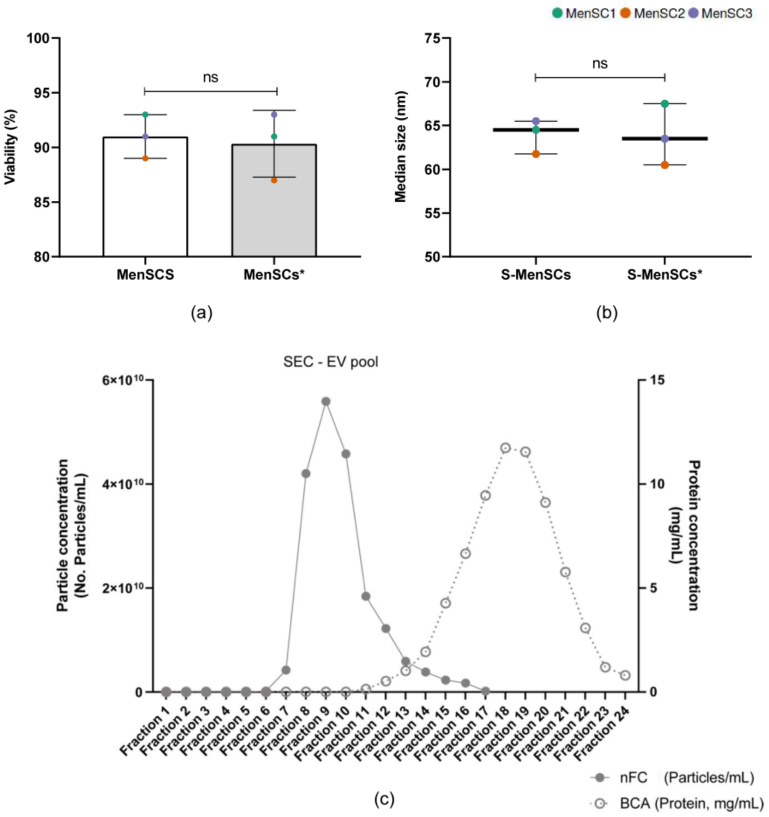

Figure S2. Additional data on MenSC-derived EVs. (a) Cell viability was checked by Trypan Blue Staining for MenSCs (withe bars) and MenSCs* (grey bars) since total cell number was used as normalizer for EV release estimation. No significant differences were found. (b) nFC analyses of total secretomes (10k × g supernatants) showed that the median size (nm) of detected particles was similar between basal (S-MenSCs) and primed samples (S-MenSCs*). Data from different donors (MenSC1, MenSC2, MenSC3) are indicated with different colors. (c) Representative plot of SEC fractions. A total of 24 fractions were collected. The left axis indicates the particle concentration detected by nFC (continuous line) and the right axis shows the protein concentration (mg/ml) detected by BCA (discontinuous line) for each fraction.

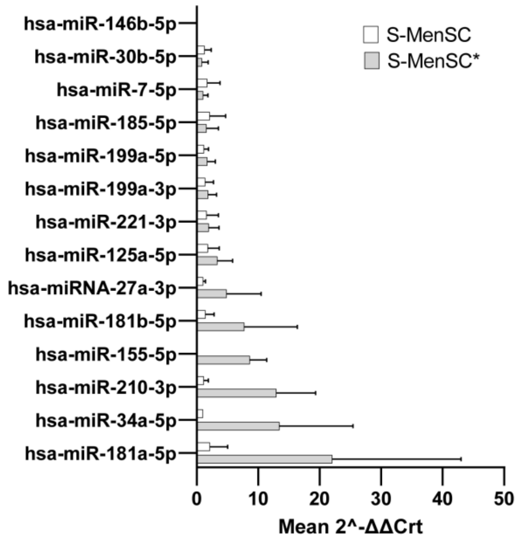

Figure S3. Confirmation of miRNA expression. A total of 14 miRNAs directly involved in the Inflammatory Response were selected to confirm the expression result of NGS analysis by qPCR. Total RNA was isolated from S-MenSCs and S-MenSCs* and the amplification products were analyzed by the $2^{-\Delta Crt}$ method using the most stables miRNAs as endogenous controls. The figure indicates the mean $2^{-\Delta\Delta Crt}$ values of miRNA expression from S-MenSCs (white bars) and S-MenSCs* (grey bars). No statistical differences were found.

Antigen	Alternative Name	Clone	Catalog number (Company)
**CD9**	p24, DRAP-1, MRP-1	M-L13	341646 (BD Pharmingen)
**CD16**	FCRIIIA, CD16a	LNK16	MCA1193F (Bio-Rad)
**CD20**	B1, Bp35	L27	335829 (BD Pharmingen)
**CD29**	Platelet GPIIa, Integrin β1, GP	4B7R	MCA1949F (Bio-Rad)
**CD38**	ADP-ribosyl cyclase, T10, Cyclic ADP-ribose hydrolase 1	AT13/5	MCA1019F (Bio-Rad)
**CD44**	ECMRII, H-CAM, Pgp-1, Phagocytic glycoprotein I, Extracellular matrix receptor III, GP90 Lymphocyte homing/adhesion receptor, Hyaluronate receptor	Clone 515	550989 (BD Pharmingen)
**CD48**	Blast-1, Hulym3, BCM-1, OX-45, MEM-102	MEM-102	MCA1053 (Bio-Rad)
**CD49a**	VLA-1α, Integrin α1	SR84	559596 (BD Pharmingen)
**CD49b**	VLA-2α, Integrin α2, gPIa	AK7	MCA743F (Bio-Rad)
**CD49c**	VLA-3α, Integrin α3, GAPB3, Galactoprotein B3, MSK18, Very Common Antigen-2 (VCA-2)	17C6	MCA1948F (Bio-Rad)
**CD49d**	VLA-4α, Integrin α4	44H6	MCA923F (Bio-Rad)
**CD49e**	VLA-5α, Integrin α5, Fibronectin receptor	JBS5	MCA1187T (Bio-Rad)
**CD49f**	VLA-6α, Integrin α6, gpI	450-30A	MCA1457F (Bio-Rad)
**CD50**	ICAM-3	ICAM3.3	MCA1485 (Bio-Rad)
**CD51**	Integrin αv, VNR-α, Vitronectin-Rα	13C2	9656-09 (Southern Biotech)
**CD54**	ICAM-1	15.2	MCA1615F (Bio-Rad)
**CD55**	Decay Accelerating Factor for Complement (DAF) S	67	MCA1614 (Bio-Rad)
**CD56**	Leu-19, NKH-1, Neural Cell Adhesion Molecule (NCAM)	B159	555516 (BD Pharmingen)
**CD58**	LFA-3	B-L28	854.632.010 (DIACLONE)
**CD59**	1F5Ag, H19, Protectin, MACIF, MIRL, P-18	BRA10G	5590-F100T (BIOCYTEX)
**CD61**	GP IIIa, Integrin β3	PM6/13	MCA728F (Bio-Rad)
**CD62E**	E-Selectin, ELAM-1, LECAM-2	CL2/6	MCA1969F (Bio-Rad)
**CD63**	LIMP, MLA1, gp55, NGA, LAMP-3, ME491, OMA81H, TSPAN30, Granulophysin, Melanoma 1 antigen	H5C6	557305 (BD Pharmingen)
**CD66a/c/e**	CD66, NCA-160, BGP (Biliary glcoprotein), BGP1, BGPI, CEACAM1, NCA, NCA-50/90,CEAL, CEACAM6	B1.1/CD66	551480 (BD Pharmingen)
**CD71**	TFRC, T9, Transferrin receptor, TFR, TRFR	DF1513	MCA1148F (Bio-RAD)
**CD73**	Ecto-5'-nuclotidase, NT5E, E5NT, NT5, NTE, eN, eNT	AD2	550257 (BD Pharmingen)
**CD90**	Thy-1	F15-42-1	MCA90F (Bio-Rad)

**CD105**	Endoglin, HHT1, ORW, SH-2	SN6	MCA1557<br>(Bio-Rad)
**CD107a**	LAMP-1, LAMPA, CD107a, LGP120	H4A3	A15798<br>(Thermo Fisher)
**CD120b**	TNFRII, p75, TNFR p80	MR2-1	MCA1944<br>(Bio-Rad)
**CD126**	IL-6Rα	M5	551850<br>(BD Pharmingen)
**CD133**	AC133, PROML1, Prominin 1, Hematopoietic stem cell antigen	293C3	130-090-853<br>(Miltenyi Biotec)
**CD152**	CTLA-4	BNI3	555853<br>(BD Pharmingen)
**CD166**	ALCAM, KG-CAM, SC-1, BEN, DM-GRASP	3A6	MCA1926F<br>(Bio-Rad)
**CD273**	B7DC, Btdc, PDL2, CD273, PD-L2, PDCD1L2, MGC142238, MGC142240, bA574F11.2, PDCD1LG2	MIH18	12-5888-73<br>(eBioscience)
**CD274**	B7-H, B7H1, PDL1, PD-L1, PDCD1L1, PDCD1LG1, MGC142294, MGC142296, CD274	MIH1	12-5589-73<br>(eBioscience)
**CD279**	PD1, CD279, SLEB2, hPD-1, hPD-l, PDCD1	MIH4	558694<br>(BD Pharmingen)
**CD282**	TIL4, CD282, TLR2	TL2.1	12-9922-42<br>(eBioscience)
**HLA-ABC**	HLA-I	G46-2.6	555553<br>(BD Pharmingen)
**HLAII DP DQ DR**	HLA-II	WR18	MCA477PE<br>(Bio-Rad)

Table S1. Panel of human monoclonal antibodies used for the phenotypic characterization by flow cytometry.

miRNAs	Accession	LogFC	*p*-Value	FDR
hsa-miR-155-5p	MIMAT0000646	5.055	7.57E-07	9.51E-05
hsa-miR-361-3p	MIMAT0004682	4.937	7.92E-04	1.99E-02
hsa-miR-376a-3p	MIMAT0000729	4.926	1.10E-05	8.73E-04
hsa-miR-424-3p	MIMAT0004749	4.757	1.17E-03	2.36E-02
hsa-miR-27a-3p	MIMAT0000084	4.349	7.64E-09	4.80E-06
hsa-miR-210-3p	MIMAT0000267	4.338	5.32E-04	1.52E-02
hsa-miR-21-3p	MIMAT0004494	4.031	3.07E-05	1.75E-03
hsa-miR-490-3p	MIMAT0002806	4.029	8.96E-04	2.08E-02
hsa-miR-26a-2-3p	MIMAT0004681	3.887	5.93E-04	1.62E-02
hsa-miR-181a-5p	MIMAT0000256	3.756	1.11E-05	8.73E-04
hsa-miR-221-5p	MIMAT0004568	3.234	2.90E-04	9.58E-03
hsa-miR-185-5p	MIMAT0000455	3.116	3.09E-03	4.73E-02
hsa-miR-34a-5p	MIMAT0000255	3.086	3.49E-05	1.82E-03
hsa-miR-181b-5p	MIMAT0000257	3.064	1.24E-04	5.20E-03
hsa-miR-30a-3p	MIMAT0000088	2.983	2.35E-04	8.21E-03
hsa-miR-221-3p	MIMAT0000278	2.804	1.33E-05	9.29E-04
hsa-miR-148b-3p	MIMAT0000759	2.787	1.39E-03	2.73E-02
hsa-miR-199a-5p	MIMAT0000231	2.754	1.10E-03	2.30E-02
hsa-miR-146b-5p	MIMAT0002809	2.585	1.04E-04	4.64E-03
hsa-miR-29c-3p	MIMAT0000681	2.560	2.02E-03	3.59E-02
hsa-miR-125a-5p	MIMAT0000443	2.381	1.06E-03	2.29E-02
hsa-miR-7-5p	MIMAT0000252	2.362	4.02E-04	1.20E-02
hsa-miR-376c-3p	MIMAT0000720	1.890	1.69E-03	3.12E-02
hsa-miR-30b-5p	MIMAT0000420	1.851	2.15E-03	3.64E-02
hsa-miR-199a-3p	MIMAT0000232	1.505	3.64E-06	3.81E-04
hsa-miR-320d	MIMAT0006764	-1.789	8.94E-04	2.08E-02
hsa-miR-4492	MIMAT0019027	-1.911	2.06E-03	3.59E-02
hsa-miR-625-3p	MIMAT0004808	-1.959	2.41E-03	3.98E-02
hsa-miR-625-5p	MIMAT0003294	-2.436	4.27E-07	6.70E-05
hsa-miR-549a-3p	MIMAT0003333	-2.505	9.58E-04	2.15E-02
hsa-miR-12136	MIMAT0049032	-2.579	4.13E-05	1.99E-03
hsa-miR-483-5p	MIMAT0004761	-2.691	3.05E-03	4.73E-02
hsa-miR-9901	MIMAT0039321	-2.710	3.23E-04	1.01E-02
hsa-miR-4444	MIMAT0018962	-2.826	1.34E-04	5.28E-03
hsa-miR-7704	MIMAT0030019	-3.075	1.96E-04	7.25E-03
hsa-miR-4516	MIMAT0019053	-3.188	2.98E-05	1.75E-03
hsa-miR-5585-3p	MIMAT0022286	-3.666	3.48E-08	1.09E-05
hsa-miR-619-5p	MIMAT0026622	-3.941	2.90E-07	6.08E-05
hsa-miR-642a-3p	MIMAT0020924	-3.943	1.47E-03	2.79E-02
hsa-miR-1-3p	MIMAT0000416	-4.093	6.45E-04	1.69E-02

Table S2. The list of the 40 significantly differentially expressed miRNAs. miRNA expression level is indicated as log fold change (logFC) between secretomes from endMSCs (n = 3) and endMSCs* (n = 3). *p* values and Benjamini-Hochberg FDR (False Discovery Rate) adjusted *p* values are shown.

miRNAs Name	Assay ID	Mature miRNA Sequence	*Inflammatory Response* GO:0006954	Other related Categories
hsa-miR-7-5p	483061_mir	UGGAAGACUAGUGAUUUUGUUGUU	✔	*Innate Immune Response* GO:0045087, *Macrophage Differentiation* GO:0030225
hsa-miR-27a-3p	478384_mir	UUCACAGUGGCUAAGUUCCGC	✔	*Innate Immune Response* GO:0045087, *T-helper 17 Cell Differentiation* GO:0072538
hsa-miR-30b-5p	478007_mir	UGUAAACAUCCUACACUCAGCU	✔	*Innate Immune Response* GO:0045087, *Macrophage Differentiation* GO:0030225
hsa-miR-34a-5p	478048_mir	UGGCAGUGUCUUAGCUGGUUGU	✔	*Innate Immune Response* GO:0045087, *Macrophage Differentiation* GO:0030225
hsa-miR-125a-5p	477884_mir	UCCCUGAGACCCUUUAACCUGUGA	✔	*Innate Immune Response* GO:0045087, *Macrophage Differentiation* GO:0030225
hsa-miR-146b-5p	483144_mir	UGAGAACUGAAUUCCAUAGGCUG	✔	*Innate Immune Response* GO:0045087
hsa-miR-155-5p	483064_mir	UUAAUGCUAAUCGUGAUAGGGGUU	✔	*Innate Immune Response* GO:0045087, *Macrophage Differentiation* GO:0030225, *T-helper 17 Cell Differentiation* GO:0072538, *T Cell Differentiation* GO0030217
hsa-miR-181a-5p	477857_mir	AACAUUCAACGCUGUCGGUGAGU	✔	*Innate Immune Response* GO:0045087, *Macrophage Differentiation* GO:0030225, *T-helper 17 Cell Differentiation* GO:0072538
hsa-miR-181b-5p	478583_mir	AACAUUCAUUGCUGUCGGUGGGU	✔	*Innate Immune Response* GO:0045087, *Macrophage Differentiation* GO:0030225
hsa-miR-185-5p	477939_mir	UGGAGAGAAAGGCAGUUCCUGA	✔	*Innate Immune Response* GO:0045087, *Macrophage Differentiation* GO:0030225, *T Cell Differentiation* GO0030217
hsa-miR-199a-3p	477961_mir	ACAGUAGUCUGCACAUUGGUUA	✔	*Innate Immune Response* GO:0045087, *Macrophage Differentiation* GO:0030225
hsa-miR-199a-5p	478231_mir	CCCAGUGUUCAGACUACCUGUUC	✔	*Innate Immune Response* GO:0045087, *Macrophage Differentiation* GO:0030225
hsa-miR-210-3p	477970_mir	CUGUGCGUGUGACAGCGGCUGA	✔	*Innate Immune Response* GO:0045087, *T-helper 17 Cell Differentiation* GO:0072538
hsa-miR-221-3p	477981_mir	AGCUACAUUGUCUGCUGGGUUUC	✔	*Innate Immune Response* GO:0045087, *Macrophage Differentiation* GO:0030225
hsa-miR-93-5p	478210_mir	CAAAGUGCUGUUCGUGCAGGUAG	✘	Suitable endogenous control
hsa-miR-16-5p	477860_mir	UAGCAGCACGUAAAUAUUGGCG	✘	Suitable endogenous control

Table S3. miRNAs name and assays ID of commercial TaqMan™ Advanced miRNA Assay (Applied Biosystems, Thermo Fisher, CA, USA), used for transcriptomic results confirmation. Additional information about their Gene Ontology (GO) Categories is also provided.
